# Development of clinically relevant QA procedures for the BrainLab ExacTrac imaging system

**DOI:** 10.1002/acm2.12301

**Published:** 2018-03-10

**Authors:** Ileana Iftimia, Per H. Halvorsen

**Affiliations:** ^1^ Radiation Oncology Department Lahey Hospital and Medical Center Burlington MA USA; ^2^ Tufts University School of Medicine Boston MA USA

**Keywords:** ExacTrac, quality assurance

## Abstract

**Purpose:**

The aim of this study was to develop Quality Assurance procedures for the BrainLab ExacTrac (ET) imaging system following the TG 142 recommendations for planar kV imaging systems.

**Materials and Methods:**

A custom‐designed 3D printed holder was used to position the Standard Imaging QCkV‐1 phantom at isocenter, facing the ET X ray tubes. The linac's light field (collimator at 45⁰) was used to position the phantom holder. The ET images were exported to ARIA where geometric distortion was checked. The DICOM images were analyzed in the PIPSpro software. The following parameters were recorded (technique 80 kV/2mAs): spatial resolution (Modulated Transfer Function (MTF) F50/F40/F30), contrast‐to‐noise ratio (CNR), and noise. A baseline was generated for future image analysis. Beam quality and exposure were measured using the Unfors R/F detector. Using a rod holder, the detector was placed at isocenter, facing each ET X‐ray tube. The measurements were performed for all preset protocols ranging from cranial low (80 kV/6.3 mAs) to abdomen high (145 kV/25 mAs). The total exposure was converted to dose.

**Results and Discussion:**

The image quality parameters were close for the two tubes. A common baseline was therefore generated. The average baseline values (both tubes, both images/tube) were 1.06/1.18/1.30, 1.32, and 67.3 for the MTF F50/F40/F30, noise, and CNR respectively. The procedure described here was used for another 24 sets. Using a positioning template and 3D printed phantom holder, experimental reproducibility has been acceptably high. The measured phantom dimensions were within 1 mm from the nominal values. The measured kV values were within 2% of the nominal values. The exposure values for the two tubes were comparable. The range of total measured dose was 0.099 mGy (cranial low) to 1.353 mGy (abdomen high).

**Conclusions:**

A reliable process has been implemented for QA of the ET imaging system by characterizing the system's performance at isocenter, consistent with clinical conventions.

## 1 INTRODUCTION

The BrainLab ExacTrac (ET) X ray 6D stereotactic IGRT system (BrainLab AG, Feldkirchen, Germany) could play an important role in image‐guided radiosurgery and radiotherapy,^1^, ^2^ on the assumption that the image quality is reliably good. This system is an integration of two subsystems: an infrared‐based optical positioning system for initial patient setup and couch movement control and a radiographic kV X ray imaging system for position verification and readjustments based on internal anatomy or implanted fiducials. This kV imaging system is configured with X ray sources in the floor and amorphous silicon flat‐panel detectors mounted near the ceiling, forming an oblique imaging geometry.

The AAPM Task Group Report 142 (TG 142)^3^ recommends checking monthly the image quality (geometric distortion, spatial resolution, contrast, uniformity, and noise) and measuring annually the beam quality/energy and imaging dose for the planar kV imaging systems. The measured values should be compared with an institution/system specific baseline generated *a priori*. The BrainLab Novalis ExacTrac system is listed in the AAPM Task Group Report 75 (TG 75)^4^ focusing on image dose management during image‐guided radiotherapy, and the dose for two extreme techniques (cranium/C‐spine and body‐thoracic/lumbar spine) are tabulated, without describing the measurement methodology.

The ET system's oblique geometry presents some challenges to conducting image quality and dose tests in the normal linear accelerator “treatment space”. Currently there is only one publication focused on specific quality assurance (QA) tests for the imaging component of the BrainLab ET system.^5^ The authors have mounted the image QA phantom and dosimeter on the surface of the image receptor panel. While that ensures consistency between measurements, we chose to design a method for reliably performing the QA tests with the phantom and dosimeter at the treatment machine's isocenter, to match the clinical settings and procedures. This study presents the QA program we developed and the tolerance values we established for the ET imaging quality parameters.

## 2 MATERIALS AND METHODS

### 2.A ExacTrac monthly quality assurance (ET MQA)

A test patient was generated in the Eclipse Treatment Planning System (Varian Medical Systems, Palo Alto, CA, USA) to be used for the ET MQA. A plan was added to the test patient containing an AP beam gantry/couch 0°, and collimator 45°.

The plan was scheduled for multiple fractions, and imported into the ET system using the reference array. An ET machine with beam energy 80 kV was generated in the PIPSpro software V 5.4 (Standard Imaging, Inc., Middleton, WI, USA). The 80 kV was selected to match the ET clinical imaging technique for the stereotactic cranial cases, also ensuring a good image quality for the phantom we used.

**Figure 1:**
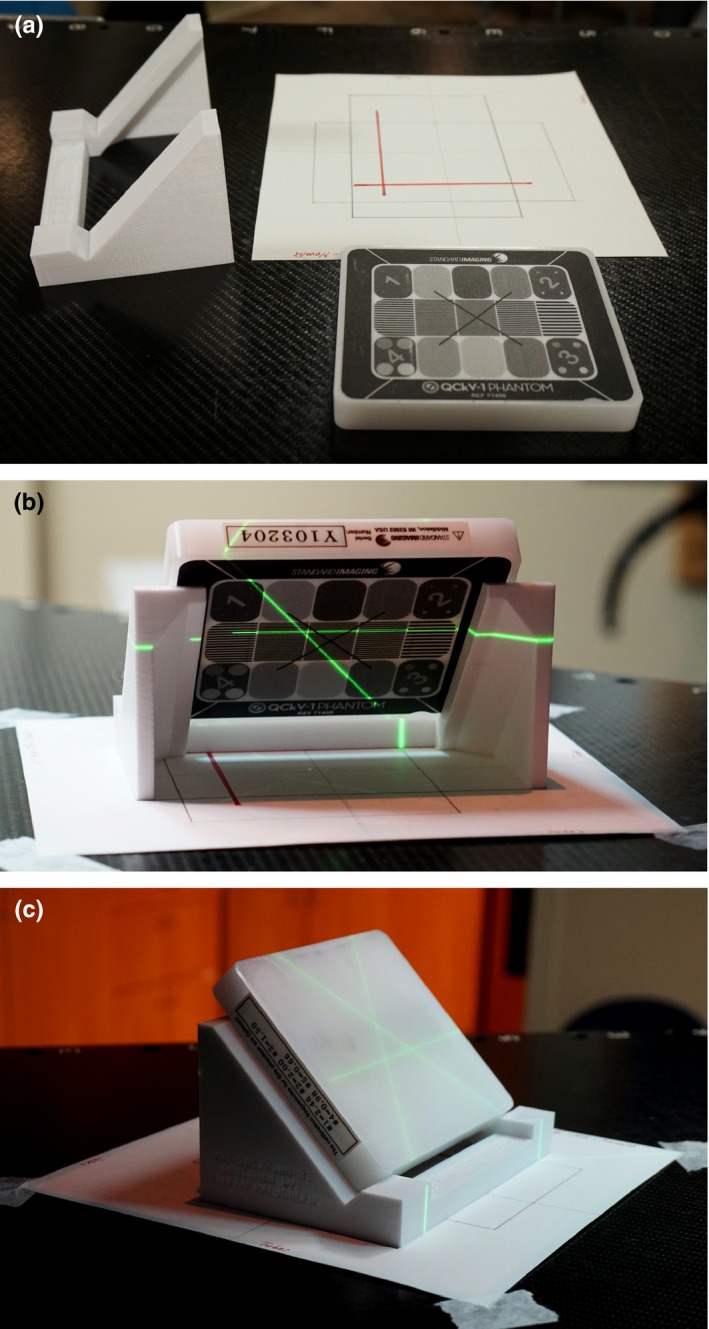
(a) Photo with the holder, phantom, and paper template used for the ET MQA testing; photo with the MQA setup for tube #2, showing laser intersection on the phantom (b) face and (c) back.

A custom‐designed 3D printed holder was used to position the Standard Imaging QCkV‐1 phantom at isocenter, facing the ET X ray tubes (i.e., rotated 45° in both horizontal and vertical planes). The linac's light field (collimator at 45°) was used to position the phantom holder. A paper template was designed for consistent phantom placement at isocenter (see Fig. 1a–c).

**Figure 2:**
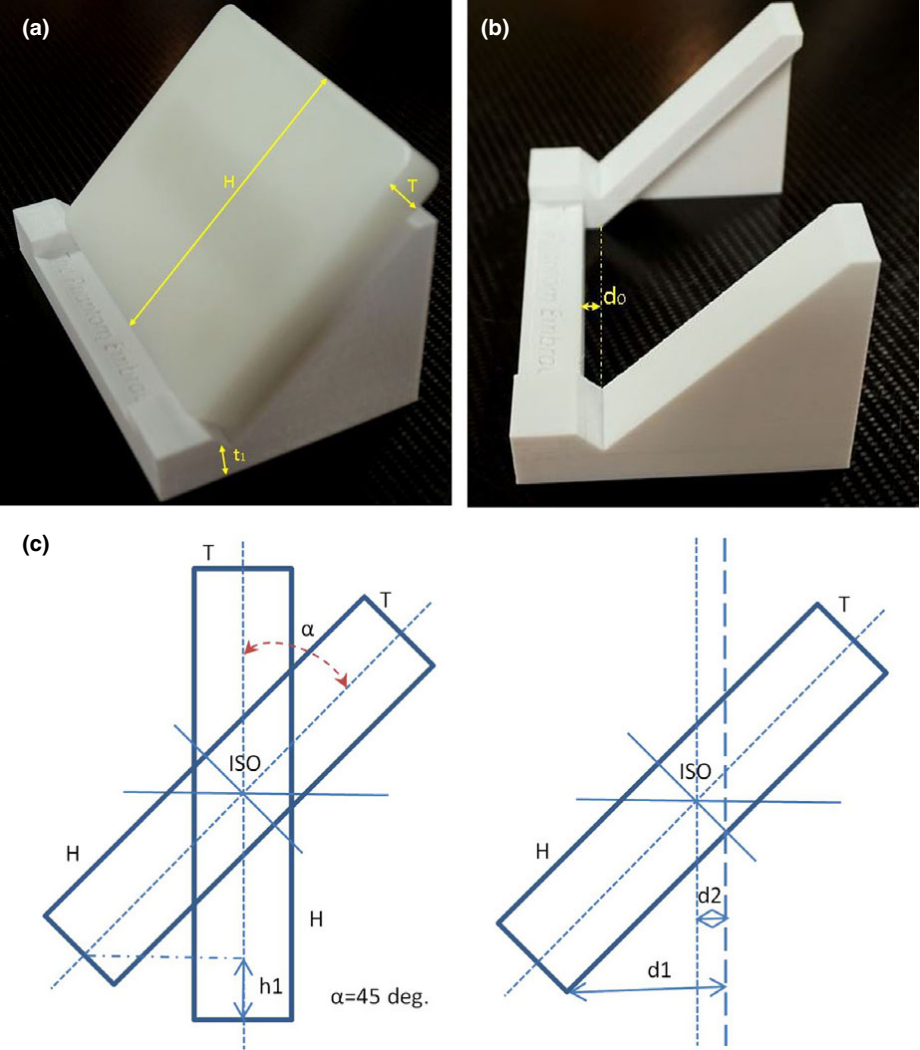
(a–c) Schematic diagrams with the holder‐phantom combination showing the distances used to estimate the couch vertical and distance from the inside bottom plate of the holder and the bottom front plane of the phantom (see text for details and notations).

To ensure the phantom on the holder is positioned at isocenter, a mathematical calculation was performed *a priori* to determine the couch vertical setting (*v*):

(1) $$V=\frac{H}{2}+t_{1}-h_{1}$$

where *H* is the phantom height, *t*_1_ is the holder thickness corresponding to the bottom side of the phantom mid‐plane, and *h*_1_ is given by:

$$h_{1}=\frac{H}{2}\left(1-\text{cos}\alpha \right)\;\left(2\right)$$

and the distance (d) from the inside part of the holder bottom plate to the laser intersection/cross‐hair is given by:

(3) $$d=d_{0}+d_{1}-d_{2}=d_{0}+\frac{H}{2}\text{sin}\alpha -\frac{T}{2}\text{cos}\alpha$$

where *d*_0_ is the distance from the inside bottom plate of the holder and the bottom front plane of the phantom and *T* is the phantom thickness (see schematic diagrams in Fig. 2a–c). The angle *α* = 45° (i.e., equal to the tilt of the ET detectors relative to a vertical axis). For the holder‐phantom combination used here the values for the “v” and “d” are 5.5 and 3.8 cm, respectively.

The reference array was attached to the Linac couch. The plan was opened on the linac and ET consoles, a pair of images (technique used: 80 kV, 2 mAs) were acquired in ET for tube #1 (Left side when facing the linac gantry, per BrainLab convention), and then the holder was repositioned to face the tube #2. Another set of two images were acquired for the second tube using the same technique, and then the AP beam was delivered. The ET images were exported in DICOM format to the ARIA Treatment Management System (Varian Medical Systems) and then from ARIA to PIPSpro. The procedure was repeated on different days to acquire five pairs of images for each tube. The images were analyzed in PIPSpro using a dual‐image approach and a baseline was generated to be used for future image analysis. The following parameters were recorded: high‐contrast spatial resolution (Modulated Transfer Function—MTF F50/F40/F30 in lp/mm), contrast‐to‐noise ratio (CNR), and noise. Tolerances for the above mentioned parameters were initially established based on our prior experience with the Varian Onboard Imaging System (Varian Medical Systems). The geometric distortion (i.e., phantom's dimension) was checked in ARIA. The approach described here was used for the ET MQA testing for over a year (14 MQA tests). Ten more tests were performed during this time to improve the statistics for data analysis. The initial PIPSpro tolerances were refined based on the results. In addition, the influence of setup errors and tube output variation on the image quality parameters was assessed.

### 2.B ExacTrac Annual Quality Assurance (ET AQA)

**Figure 3:**
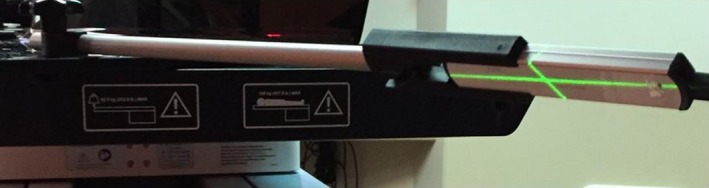
Photo showing the setup for the ET AQA testing.

The beam quality/energy and exposure were measured using the Unfors RaySafe Xi R/F detector (*Unfors RaySafe AB, Billdal, Sweden*). Using a rod holder, the detector was placed at isocenter, facing in turn each ET X ray tube (see Fig. 3). The gantry and couch were set to 0°. The collimator was set to 45°, such that the light field projection could help to set the detector at isocenter, rotated 45° relative to the couch long axis in a horizontal plane. After that, the rod with the detector attached was rotated 45° relative to a vertical axis passing through the isocenter (i.e., facing an X ray tube). The measurements were performed for all preset protocols ranging from cranial low (80 kV/6.3mAs) to abdomen high (145 kV/25mAs), exposing only one tube at a time and re‐orienting the detector for the second tube. The kV was recorded for each tube and compared with the nominal values. The total exposure (summed for both tubes) was converted to dose assuming an exposure to “in‐air” dose conversion factor of 0.87. For the ET energy range (80–145 kV) the exposure‐to‐dose conversion factor may be about 8–10% higher for water (tissue equivalent) media,^6^ consequently patient dose could be somewhat larger than estimated in this work. Setup errors and tube output fluctuations were assessed to evaluate the uncertainties of the measured dose and kV values.

## 3 RESULTS AND DISCUSSION

### 3.A ET MQA

Five pairs of images were initially acquired for each tube and analyzed. The image quality parameters were close for the two tubes, consequently a common database was generated in PIPSpro. The procedure described above was used for 24 sets (see Section 2.A). Table 1 shows the average image quality parameters and range (% difference minimum and maximum values versus average) for the tube #1, while Table 2 shows the average image quality parameters for both tubes, both images, compared to the baseline values. Table 2 also shows the tolerances set in PIPSpro. The CNR value seems to be somewhat higher for tube #1 (tube #1: average CNR ~70; tube#2: average CNR ~64), which is reflected in a slight increase in MTF F50 for this tube. Also, the noise and CNR have somewhat less fluctuation for tube #1. On the basis of this analysis, we decided to change the MTF tolerances in PipSpro from the initial values of 10% for “warning”, 15% for “fail” to 5% for “warning” and 10% for “fail”. The noise and CNR have more fluctuation than MTF, consequently we decided to keep the initial tolerances for those parameters.

**Table 1:** Results for tube 1 (average over 24 sets)

	Image 1					Image 2			
	Noise both img.	F50	F40	F30	CNR	F50	F40	F30	CNR
Average	1.25	1.07	1.19	1.31	70.3	1.07	1.19	1.31	70.4
%Diff. min vs average	−16	−0.9	−0.7	−0.7	−4.7	−1.1	−0.8	−0.6	−4.7
%Diff. max vs average	12	3.2	1.7	0.6	3.0	3.5	1.7	0.4	3.2

**Table 2:** Baseline values and results for both tubes (average over 24 sets)

	Noise	F50	F40	F30	CNR
Baseline values (five initial sets, for each tube)	1.32	1.06	1.18	1.30	67.30
Average (both tubes; both img., 24 sets)	1.29	1.07	1.19	1.31	66.90
%diff. av. vs baseline	−2.0	0.5	0.6	0.7	−0.6
Refined tolerances (see text for details)	±20% warning; ±25% fail	±5% warning; ±10% fail	±5% warning; ±10% fail	±5% warning; ±10% fail	±15% warning; ±20% fail

Standard Imaging's recommendation is to use the QCkV‐1 phantom only for the range 70–100 kV, with a low mAs. We performed measurements at four kV values (range 50–110), but for simplicity we decided to use a single setup (80 kV/2 mAs) for our routine MQA tests. Currently, we use the ET system primarily for cranial cases (kV 80–100) and occasionally for Head and Neck cases (kV 100). If the ET system will be used for pelvic cases (kV 145), a different phantom may be needed in order to test the image quality for that energy.

Table 3 shows for comparison the noise, MTF, and CNR values for 100 kV/1 mAs technique, averaged for five sets over both tubes, both images. For 100 kV, we had to lower the mAs to 1, otherwise the image becomes too dark and cannot be analyzed using the procedure described here.

**Table 3:** Image quality parameters (average over five tests) for the 100 kV/1 mAs technique

	Noise	F50	F40	F30	CNR
Baseline values (five initial sets, for each tube for 80 kV/2mAs)	1.32	1.06	1.18	1.30	67.30
Average (both tubes; both img., five sets) for 100 kV/1mAs	1.47	1.06	1.19	1.31	57.50
%Diff. av. vs baseline at 80 kV/2 mAs	<11.5	<0.5	<1.0	<1.0	<−15.0

The MTF values were very close to the ones for the 80 kV/2mAs technique, but the noise increased and consequently the CNR decreased. This may be correlated with the lower mAs used for this higher energy. For the cranial and head and neck clinical cases imaged with 80–100 kV, the mAs is in the range 6.3–12.5, therefore the noise would be reduced comparative to our in‐phantom measurements. Furthermore, for patients the contrast level is adjusted as needed, while for our measurement we kept that at its lowest position for setup reproducibility.

The image quality parameters were similar for the measurements performed in the same day, keeping the same setup or redoing it, and for those performed months later. Both the setup errors and the tube output fluctuations contribute to the variation in image quality parameters, but the former contribution seems minimal.

**Figure 4:**
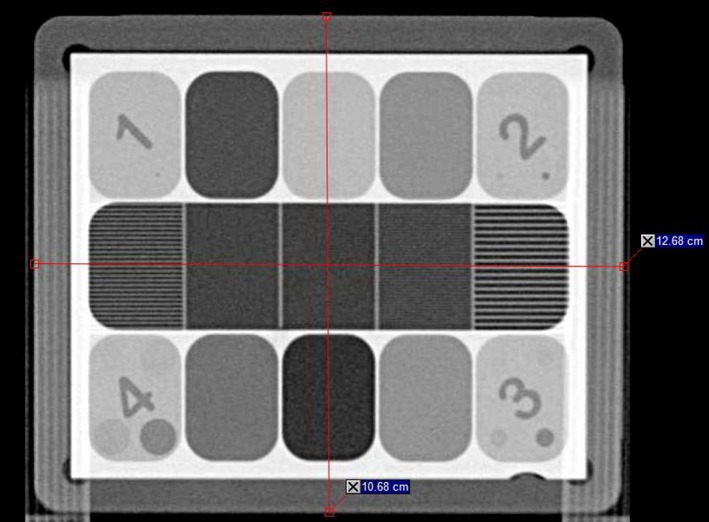
Phantom image imported in ARIA with dimensions displayed.

Figure 4 shows a phantom image acquired as described above and imported to ARIA in DICOM format. The phantom dimensions (i.e., geometric distortion) as measured on the image (12.68 × 10.68 mm^2^) are within 1 mm from the nominal phantom dimensions (12.70 × 10.75 mm^2^).

### 3.B ET AQA

Two AQA tests were performed to date using the measurement approach described above (ET system version 6.2.0.). Table 4 gives the results for the latest AQA test and the %difference for the total dose relative to the prior AQA values. The measured kV values for each tube were within 2% from the nominal values. The variation in dose from 1 year to another was within ~2.5%. These variations are in agreement with the typical 5% tolerance for the kV and dose measurements in diagnostic imaging, assuming the same X ray tube, filtration, and setup. The dose values exhibit a linear dependence on mA and a ~quadratic dependence on kV. The range of total measured dose was 0.099 mGy (cranial low) to 1.353 mGy (abdomen high). The total measured dose for the cranium high protocol (0.323 mGy) was in good agreement with the cranium/C spine dose value of 0.335 mGy listed in the AAPM TG 75 report.^4^

**Table 4:** AQA results for the ET system version 6.2.0 (kV and dose)

Protocol	Technique kV/mAs	Meas. kV tube 1	%Diff. from nominal value	Meas. kV tube 2	%diff. from nominal value	Total dose (mGy)	%diff. from prior AQA
Abdomen high	145/25	146.0	0.7	143.0	−1.4	1.353	1.7
Cranial high	100/12.5	100.1	0.1	99.8	−0.2	0.323	2.4
Cranial low	80/6.3	80.8	1.0	80.3	0.4	0.099	1.7
Head and neck high	100/10	100.4	0.4	99.3	−0.7	0.258	2.0
Pelvis high	130/25	132.1	1.6	129.4	−0.5	1.104	1.8
Thorax medium	120/25	121.9	1.6	120.1	0.1	0.952	2.5

Accurate alignment of the Unfors detector with the ET X ray beam is critical for accurate measurement of kV. The current method of rotating the rod holder is limited in precision due to the lack of an angle indicator. We are in the process of redesigning the rod holder setup in order to increase its consistency.

## 4 CONCLUSIONS

A reliable methodology has been implemented for QA of the ET imaging system by characterizing the system's performance at isocenter, in agreement with clinical conventions. The ExacTrac system showed stable image quality parameters (high‐contrast spatial resolution, CNR, and geometrical accuracy) and dose/kVp over a year. The parameters were comparable for the two tubes, consequently a common baseline could be generated for the MQA testing. Separate baselines may be considered for the two tubes in order to tighten the tolerances further. Based on individual tube image test results to date the warning/fail thresholds can be set to 3%/5% for the MTF and to 10%/15% for the CNR. The low mAs values necessary for these tests may be responsible for the level of noise/noise fluctuation. Similar with clinical procedure, we run the beams for both tubes simultaneously, and then we analyze in turn the image for the tube facing the phantom. The scatter radiation from one tube may contribute to the noise fluctuation for the other one, though a simple test with one tube covered by 1.0 mm lead did not show a significant difference.

For the MQA, future work will focus on the PIPSpro “single image method”, requiring one phantom and one flood field image. In addition to image quality parameters as currently obtained via the dual‐image method, this method will provide uniformity calculations, per the AAPM TG 142 recommendations.

For the AQA, future work will focus on rod holder setup redesign to improve consistency, avoiding variation in the attenuation/scatter from the detector lead back coating.

## CONFLICT OF INTEREST

None.
